# Therapeutic monitoring of amiodarone and desethylamiodarone after surgical ablation of atrial fibrillation-evaluation of the relationship between clinical effect and the serum concentration^☆^

**DOI:** 10.1016/j.jsps.2021.03.004

**Published:** 2021-03-31

**Authors:** Erika Hrudikova, Milan Grundmann, Martin Kolek, Romana Urinovska, Ivana Kacirova

**Affiliations:** a Department of Clinical Pharmacology, Faculty of Medicine, University of Ostrava, Syllabova 19, 703 00 Ostrava, Czech Republic; b Department of Clinical Pharmacology, Department of Laboratory Medicine, University Hospital Ostrava, 17. Listopadu 1790, 708 52 Ostrava, Czech Republic; c Department of Clinic Subjects, Faculty of Medicine, University of Ostrava, Syllabova 19, 703 00 Ostrava, Czech Republic; d Department of Cardiac Surgery, University Hospital Ostrava, 17. Listopadu 1790, 708 52 Ostrava, Czech Republic

**Keywords:** Amiodarone, Atrial fibrillation, Desethylamiodarone, Maze procedure, Serum concentration, Sinus rhythm, AMI, amiodarone, AF, atrial fibrillation, ALT, alanine transaminase, AST, aspartate transaminase, CABG, coronary artery bypass graft, DEA, desethylamiodarone, ECG, electrocardiogram, GMT, gama glutamyl transferase, SA, surgical ablation, SR, sinus rhythm, ST, supraventricular tachyarrhythmia, TDM, therapeutic drug monitoring, TSH, thyroid-stimulating hormone

## Abstract

**Background:**

Association between clinical effect and serum concentration of amiodarone (AMI) and its active metabolite desethylamidarone (DEA) in patients after surgical ablation (SA) of atrial fibrillation (AF) has not yet been studied.

**Aims:**

We wanted to find a correlation between AMI and DEA serum concentration and maintaining sinus rhythm (SR) after SA of AF.

**Methods:**

Sixty eight patients with AF who had undergone surgical ablation between 2014 and 2017 were included in a single-centre, prospective, observational study. Maintaining of SR was evaluated by standard 12-lead ECG and 24-hour Holter ECG monitoring at months 1, 3, 6 and 12 following surgery. Therapeutic monitoring of AMI and DEA concentrations was done to optimize therapy and adverse effects were followed up.

**Results:**

We have noticed a high success rate in maintaining of SR (overall 83%). The median of serum concentration of AMI was 0.81 mg/L (range 0.16–2.35 mg/L) and DEA 0.70 mg/l (range 0.19–2.63 mg/L). No significant differences were found in the serum concentratration of AMI, DEA or DEA/AMI concentratration ratios between patients with SR and persistent supraventricular tachyarrhythmia except on the second outpatient visit. We observed significant correlation between serum concentration of DEA and thyroid-stimulating hormone elevation.

**Conclusion:**

We confirmed the efficacy of AMI and DEA at the measured serum concentrations. However, analysis of these concentrations alone cannot replace assessment of the clinical response for treatment. Establishment of individual AMI (and DEA) concentrations at which the optimal therapeutic response is achieved seems to be advantageous. Therapeutic monitoring of AMI and DEA is helpful in personalised pharmacotherapy after SA of AF.

## 1 Introduction

Surgical ablation (SA) is an effective treatment option for patients with atrial fibrillation (AF) (Calkins et al., 2017). Cox (Cox et al., 2018) first introduced SA of AF as the so-called Maze I procedure. Through ongoing investigation and optimization, the method evolved into the Maze III procedure and it became the gold standard of AF surgical treatment. SA of AF is most commonly applied as a concomitant procedure during valve or coronary artery bypass graft (CABG) surgeries. The large left atrium, non-paroxysmal AF, duration of AF, advanced age and failure to isolate the entire posterior left atrium are common predictors of reduced long-term efficacy of SA (Calkins et al., 2018. 2017, Kirchhof et al., 2016). Combining of SA of AF with antiarrhythmic drugs (i.e. ‘hybrid’ rhythm control therapy) seems reasonable for AF treatment, although there is little evidence from controlled trials supporting its use. Antiarrhythmic drug therapy is commonly used for 8–12 weeks after ablation to reduce early recurrences of AF (Kirchhof et al., 2016). Amiodarone (AMI) is the most effective antiarrhythmic medication available today for the treatment of both atrial and ventricular arrhythmias (Kumar and Zimetbaum, 2013). The recommended daily dose for maintaining sinus rhythm is 200 mg once daily (Kirchhof et al., 2016). Unfortunately, AMI is able to interact with a number of other drugs and its unusual pharmacokinetics result in interindividual variation of its serum level concentrations (Hrudikova Vyskocilova et al., 2017). Therapeutic drug monitoring (TDM) of both AMI and its pharmacological active metabolite desethylamidarone (DEA) can contribute to determining their optimal concentration for individual patients. However, association between AMI and DEA serum concentration and their clinical efficacy is still difficult to evaluate. Although several studies have shown that AMI (and DEA) serum concentration in a range between 0.5 and 2.5 mg/L appears to be the most effective (Mostow et al., 1984, Mostow et al., 1986, Rotmensch et al., 1984, Tieleman et al., 1997), others demonstrate no difference between responders and non-responders in terms of serum concentrations (Greenberg et al., 1987, Haffajee et al., 1983, Rotmensch et al., 1983, Saksena et al., 1983). The purpose of this study was to assess the correlation between AMI and DEA serum concentration and maintaining sinus rhythm (SR) in patients after SA of AF.

## 2 Method

### 2.1 Study design and data acquisition

The current study was a single-centre, prospective, observational study which comprised the data of 68 patients (30 females and 38 males) with paroxysmal, persistent or longstanding persistent AF who had undergone SA between October 2014 and October 2017 at Department of Cardiac Surgery, University Hospital Ostrava, Czech Republic. All patients underwent combined heart surgery, a part of which was the left atrial cryoablation in the right-side and left-side pulmonary veins (PVs) isolation, box lesion (connecting right and left PVs lesions), lesion of the left atrium isthmus (connecting lesion with mitral annulus), resection of the left atrial appendage, and connecting lesion of the appendage base with left superior PV. The cardioblate Cryoflex probe (The Cardioblate CryoFlex Argon-powered Cryoablation System, Medtronic USA, Inc.) was used (Barta and Brat, 2017).

Patients were followed up in an out-patient clinic approximately 1, 3, 6 and 12 months after surgery. Maintaining of SR was evaluated by a 12-lead electrocardiogram (ECG) (at the first visit) and 24-hour Holter ECG monitoring and 12-lead ECG (at other visits). Any episode lasting more than 30 s was considered a recurrence of AF. Trough (before administration) blood samples of AMI (and DEA) were taken and routine TDM was used to optimize drug dosage. In addition to baseline data including a renal function test (serum creatinine and urea), liver function test - alanine transaminase (ALT), aspartate transaminase (AST) and gama glutamyl-transferase (GMT), and thyroid-stimulating hormone (TSH) were obtained. If persistent supraventricular tachyarrhythmia (atrial fibrillation, atrial flutter, supraventricular tachycardia) was present, a recommendation to escalate antiarrhythmic therapy according to TDM was applied. Direct current (DC) cardioversion was performed when SR was still not reached. The antiarrhythmic therapy was discontinued at 3–6 months postoperatively in the event of a stable SR, verified by a 24-hour Holter ECG monitoring (according to attending cardiologist).

### 2.2 Patients’ characteristics

The basic characteristics and medication used before surgery are shown in Table 1, Table 2. The spectrum of primarily indicated cardiosurgical procedures is shown in Table 3.

**Table 1:** The basic characteristics of the patients.

gender	30 female/38 male
age (mean ± SD; range)	71 ± 6; (55–82) years
weight (mean ± SD; range)	85 ± 16; (52–119) kg
height (mean ± SD; range)	168 ± 10; (145–188) cm
AMI treatment before SA of AF	15 patients
duration of persistent AF (mean ± SD; range)	4.3 ± 2.1; (0.5–9) months
duration of longstanding persistent AF (range):	
1–5 years	14
>5 years	1
>10 years	1
type of AF:	
paroxysmal	26 patients
persistent	25 patients
longstanding persistent	17 patients
heart rhythm at admission to SA of AF:	
SR	32 patients
supraventricular tachyarrhythmia*	36 patients
heart rhythm at dismission:	
SR	59 patients
supraventricular tachyarrhythmia*	9 patients
implantable cardioverter-defibrillator before/after SA	2/0 patients
permanent pacemaker before/after SA	2/15+ patients
out-patient clinic visits:	
1st visit	90% of patients of which 100% with AMI
2nd visit	84% of patients of which 91% with AMI
3th visit	81% of patients of which 58% with AMI
4th visit	76% of patients of which 29% with AMI
duration of follow-up (mean ± SD; range)	192 ± 116 (20–391) days
overall mortality	7 patients

*supraventricular tachyarrhythmia = atrial fibrillation, atrial flutter, supraventricular tachycardia

+ newly implanted dual chamber pacemakers

**Table 2:** **a)-f)** Other medications.

(a) Medication-used before surgery (68 patients)		
drug	number of patients	%
beta-blockers	57	84
ACE inhibitors or AT1 blockers	52	76
diuretics	47	69
hypolipidemics	42	62
anticoagulants and/or antiplatelet drugs	39	57
calcium blockers	21	31
antidiabetics	16	24
antiasthmatic drugs	10	15
other antihypertensives	7	10
other drugs	55	81

(b) Medication at discharge (68 patients)		
drug	number of patients	%
anticoagulants and/or antiplatelet drugs	67	99
beta-blockers	43	63
diuretics	42	62
hypolipidemics	41	60
ACE inhibitors or AT1 blockers	39	57
antidiabetics	16	24
calcium blockers	14	21
antiasthmatic drugs	11	16
other antihypertensives	2	3
other drugs	65	96

(c) Medication at first out-patient visit (53 patients)		
drug	number of patients	%
anticoagulants and/or antiplatelet drugs	52	98
beta-blockers	42	79
diuretics	38	72
ACE inhibitors or AT1 blockers	33	62
hypolipidemics	32	60
calcium blockers	14	26
antidiabetics	12	23
antiasthmatic drugs	8	15
other antihypertensives	1	2
other drugs	49	92

(d) Medication at second out-patient visit (44 patients)		
drug	number of patients	%
anticoagulants and/or antiplatelet drugs	42	95
beta-blockers	38	86
diuretics	33	75
hypolipidemics	31	70
ACE inhibitors or AT1 blockers	29	66
antidiabetics	12	27
calcium blockers	11	25
antiasthmatic drugs	8	18
other antihypertensives	1	2
other drugs	36	82

(e) Medication at third out-patient visit (31 patients)		
drug	number of patients	%
anticoagulants and/or antiplatelet drugs	31	100
beta-blockers	26	84
hypolipidemics	25	81
ACE inhibitors and/or AT1 blockers	24	77
diuretics	24	77
calcium blockers	11	35
antidiabetics	9	29
antiasthmatic drugs	2	6
other antihypertensives	1	3
other drugs	23	71

(f) Medication at fourth out-patient visit (11 patients)		
drug	number of patients	%
beta-blockers	11	100
anticoagulants and/or antiplatelet drugs	10	91
hypolipidemics	10	91
ACE inhibitors or AT1 blockers	9	82
diuretics	9	82
antidiabetics	4	36
calcium blockers	1	9
other antihypertensives	1	9
antiasthmatic drugs	0	0
other drugs	10	91

**Table 3:** Surgical procedures.

Procedure	Number of patiens (n = 68)	%
MV repair	1	1.5
MV repair + TV repair	15	22.1
MV repair + TV repair + AV replacement	2	2.9
MV repair + TV repair + ASD/PFO	3	4.4
MV repair + AV replacement	2	2.9
MV repair + AV replacement + epicardial left ventricular electrode	1	1.5
MV repair + CABG	5	7.4
MV repair + CABG + TV repair	3	4.4
MV repair + CABG + TV repair + PFO	1	1.5
MV repair + CABG + AV replacement	2	2.9
MV repair + CABG + AV replacement + PFO	1	1.5
MV repair + CABG + PFO	1	1.5
MV repair + epicardial left ventricular electrode	1	1.5
MV replacement	5	7.4
MV replacement + TV repair	2	2.9
MV replacement + AV replacement	1	1.5
MV replacement + CABG	2	2.9
MV replacement + CABG + AV replacement	1	1.5
CABG	8	11.8
CABG + TV repair	1	1.5
CABG + AV replacement	3	4.4
CABG + CEA	1	1.5
AV replacement	3	4.4
AV replacement + PFO	1	1.5
Bentall	1	1.5
TV repair	1	1.5

ASD = atrial septal defect, AV = aortic valve, Bentall = Bentall operation, CABG = coronary artery bypass grafting, CEA = carotid endarterectomy, MV = mitral valve, PFO = patent foramen ovale, TV = tricuspid valve

### 2.3 Ethical approval

An independent ethics committee (The Ethics Committee of University Hospital Ostrava, Czech Republic) approved the clinical study protocol (Reference number 656/2014). Informed consent was obtained from participants prior to study commencement.

### 2.4 Assay method

The AMI and its major metabolite DEA concentrations were determined in serum by liquid chromatography using UV/VIS detection at 240 nm. Chromatographic separation was carried out on a Kinetex PFP column using isocratic elution mobile phase of methanol:triethylamine :acetic acid (99.942:0.008:0:05,v/v/v). The pH of the mobile phase was adjusted to 4.7 with phostoric acid. Sample preparation consisted of extraction into ether. 100 µL of serum was extracted to 1 mL ether in the presence of 100 µL 1 mol/L sodium dihydrogen phosphate. After centrifugation the upper ether layer was transferred into a clean vial and evaporated to dryness. Residue was dissolved in 200 µL methanol and 10 µL was used for the analysis.

The method was validated according to FDA rules. Linearity was between 0.25 and 8.0 mg/L for both AMI and DEA and the within-day and between-day precision and accuracy were studied at three concentration levels. At tested concentrations recovery was 92.8–111.4% and the coefficient of variations were 3.4–7.4%, respectively. The LOQ was 0.25 mg/L for AMI and DEA and LOD 0.15 mg/L for both drugs. The method was quality controlled by external quality control (EQC) RfB (Bonn, Germany) twice a year.

## 3 Statistical analysis

AMI and DEA serum concentrations were used for the assessment of the sum of AMI + DEA concentrations and the ratio of DEA/AMI concentration. Statistical analysis was carried out with GraphPad Prism version 5.00 for Windows, GraphPad Software (San Diego, CA, USA; www.graphpad.com). The D’Agostino and Pearson omnibus normality test was used as first for test if the values come from a Gaussian distribution. Thereafter, the unpaired *t* test (when the values follow the Gaussian distribution; expressed as mean ± SD) or the nonparametric Mann–Whitney test (in reason of negative Gaussian distribution; expressed as median with range) were used for comparison of distributions of two unmatched groups. Similarly, the Pearson correlation test (for the Gaussian distribution) or the Spearman nonparametric correlation test were used for the correlation analysis. A p value of <0.05 was considered statistically significant.

## 4 Results

**Fig. 1:**
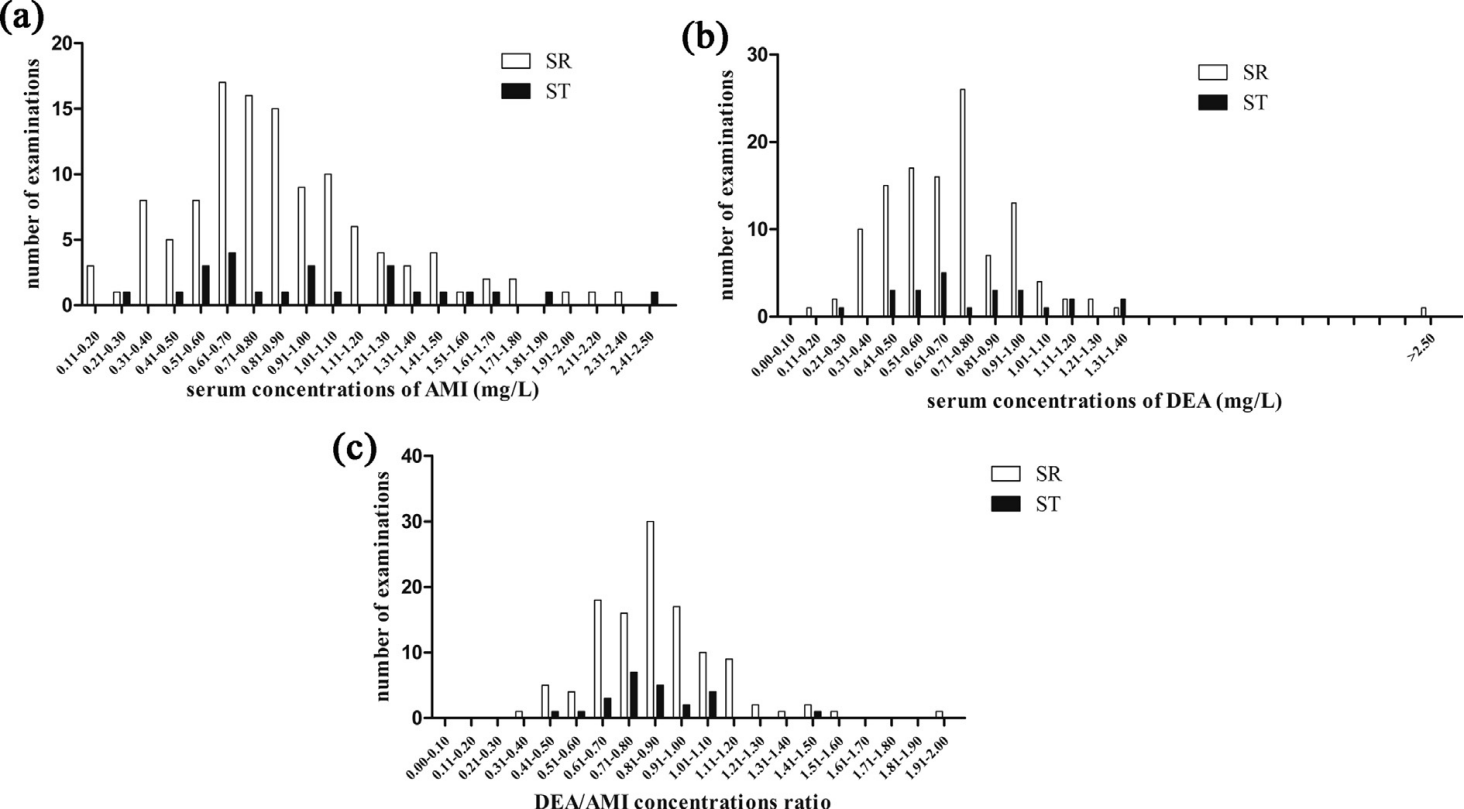
**a-c)** Distribution of all amiodarone (AMI) and desethylamiodarone (DEA) serum concentrations and DEA/AMI concentration ratio. (a) Distribution of all AMI serum concentrations. (b) Distribution of all DEA serum concentrations. (c) Distribution of all DEA/AMI concentration ratio.

The serum concentration of AMI and DEA, sum of AMI + DEA concentration, DEA/AMI concentration ratio and dose of AMI for each individual visit (and also during total time) are shown in Table 4. Distribution of all AM and DEA serum concentrations and DEA/AMI concentration ratios are shown in Fig. 1a-c.

**Table 4:** Comparison of dosage, AMI and DEA serum concentrations, sum of AMI + DEA concentrations and DEA/AMI concentrations ratio in patients with sinus rhythm (SR) and persistent supraventricular tachyarrhythmia (ST) N…number of patients, M…number of measurements.

**1st out-patient visit**							
**SR** **(N = 45)**		**Dose (mg/day)**	**Dose** **(mg/kg)**	**AMI** **(mg/L)**	**DEA** **(mg/L)**	**AMI + DEA (mg/L)**	**DEA/AMI** **ratio**
	median mean SD min max	300 268 94 143 600	3.19 3.27 1.04 1.93 7.06	0.71 0.77 0.40 0.16 2.35	0.60 0.65 0.35 0.31 2.63	1.27 1.42 0.71 0.47 4.98	0.87 0.92 0.29 0.38 1.94
**ST** **(N = 9)**	median mean SD min max	200 318 357 143 1200	2.59 4.30 4.96 1.28 16.44	0.66 0.68 0.22 0.29 1.03	0.59 0.58 0.15 0.3 0.81	1.18 1.25 0.36 0.59 1.84	0.86 0.89 0.11 0.73 1.03
*p value*		*0.1679*	*0.4248*	*0.5876*	*0.6053*	*0.5526*	*0.8360*


**2nd out-patient visit**							

**SR** **(N = 39)**		**Dose (mg/day)**	**Dose** **(mg/kg)**	**AMI (mg/L)**	**DEA (mg/L)**	**AMI + DEA (mg/L)**	**DEA/AMI** **ratio**
	median mean SD min max	300 261 69 143 400	3.33 3.15 0.86 1.43 4.41	0.81 0.81 0.36 0.16 1.98	0.74 0.67 0.23 0.19 1.12	1.58 1.49 0.57 0.35 2.93	0.85 0.88 0.20 0.48 1.44
**ST** **(N = 6)**	median mean SD min max	300 317 75 200 400	3.77 3.92 0.88 3.03 5.56	1.51* 1.54 0.51 0.9 2.42	1.03* 1.02 0.25 0.67 1.38	2.56* 2.56 0.76 1.58 3.8	0.66* 0.67 0.07 0.57 0.76
*p value*		*0.0991*	*0.1229*	*0.0013*	*0.0148*	*0.0028*	*0.0059*


**3th out-patient visit**							

**SR** **(N = 24)**		**Dose (mg/day)**	**Dose** **(mg/kg)**	**AMI (mg/L)**	**DEA (mg/L)**	**AMI + DEA (mg/L)**	**DEA/AMI** **ratio**
	median mean SD min max	300 265 66 171 400	3.17 3.15 0.67 1.94 4.41	0.97 1.03 0.32 0.62 1.71	0.81 0.82 0.22 0.44 1.32	1.80 1.85 0.50 1.18 3.03	0.85 0.82 0.18 0.42 1.11
**ST** **(N = 7)**	median mean SD min max	300 274 90 143 400	3.73 3.51 0.72 2.46 4.17	0.93 0.89 0.36 0.47 1.37	0.75 0.84 0.320.46 1.38	1.68 1.73 0.66 1.07 2.75	0.98 0.98 0.23 0.75 1.43
*p value*		*0.7866*	*0.2284*	*0.2877*	*0.803*	*0.6168*	*0.0506*


**4th out-patient visit**							

**SR** **(N = 9)**		**Dose (mg/day)**	**Dose** **(mg/kg)**	**AMI** **(mg/L)**	**DEA** **(mg/L)**	**AMI + DEA (mg/L)**	**DEA/AMI** **ratio**
	median mean SD min max	300 260 67 143 350	3.16 3.25 0.62 2.31 4.29	1.10 1.18 0.49 0.37 2.17	0.79 0.83 0.27 0.29 1.23	1.83 2.01 0.75 0.66 3.4	0.75 0.72 0.09 0.57 0.86
**ST** **(N = 2)**	median mean SD min max	400 400 283 200 600	5.29 5.29 4.82 1.89 8.70	1.54 1.54 0.38 1.27 1.81	0.93 0.93 0.09 0.86 0.99	2.47 2.47 0.29 2.26 2.67	0.63 0.63 0.22 0.48 0.79
*p value*	**	**	**	**	**	**	

**Total**							
**SR** **(M = 117)**		**Dose (mg/day)**	**Dose** **(mg/kg)**	**AMI (mg/L)**	**DEA (mg/L)**	**AMI + DEA (mg/L)**	**DEA/AMI** **ratio**
	median mean SD min max	300 264 78 143 600	3.19 3.21 0.87 1.43 7.06	0.81 0.87 0.40 0.16 2.35	0.70 0.70 0.29 0.19 2.63	1.50 1.57 0.65 0.35 4.98	0.85 0.87 0.24 0.38 1.94
**ST** **(M = 24)**	median mean SD min max	250 313 225 143 1200	3.46 4.07 3.14 1.28 16.44	0.94 1.03 0.51 0.29 2.42	0.73 0.79 0.29 0.30 1.38	1.67 1.82 0.77 0.59 3.80	0.81 0.84 0.20 0.48 1.43
*p value*	*0.8782*	*0.4342*	*0.2150*	*0.1460*	*0.2090*	*0.4733*	

* patients with sinus rhythm versus patients with supraventricular tachyarrhythmia (compared by nonparametric Mann–Whitney test)

**statistic analysis was not performed (small number of patients)

Holter ECG monitoring was carried out in 97% of all follow-up examinations of patients. SR had been detected in 83% of all measurements whereby for these measurements, the median of serum concentration of AMI was 0.81 mg/L (range 0.16–2.35 mg/L) and of DEA it was 0.70 mg/L (range 0.19–2.63 mg/L). Any significant difference was not found between two groups (patients with SR versus patients with supraventricular tachyarrhythmia) in dosage, AMI and DEA serum concentrations, sum of AMI + DEA concentrations and DEA/AMI concentrations ratio with the exception of the second outpatient visit, in which AMI, DEA and the sum of AMI + DEA concentrations were significantly higher in patients with persistent supraventricular tachyarrhythmia (table 4). Simultaneously, the DEA/AMI concentration ratio was significantly lower in this patient subgroup.

SR was detected in 83% of patients on the first visit, in 87% of patients at the second visit, in 77% of patients at the third visit and in 82% of patients at the final visit, if AMI was used. On the other hand, overall, 91% of patients with currently present supraventricular tachyarrhythmia suffered from non-paroxysmal AF at admission to heart surgery. DC cardioversion was performed in 5 patients at the first visit (SR was reached in 4 patients); in 4 patients at the second visit (SR was reached in all patients); in 3 patients at the third visit (SR was reached in 2 patients) and in 1 patient at the fourth visit (SR was reached). One patient required an implantable cardioverter defibrillator (ICD) and 15 patients required placement of a permanent pacemaker.

**Fig. 2:**
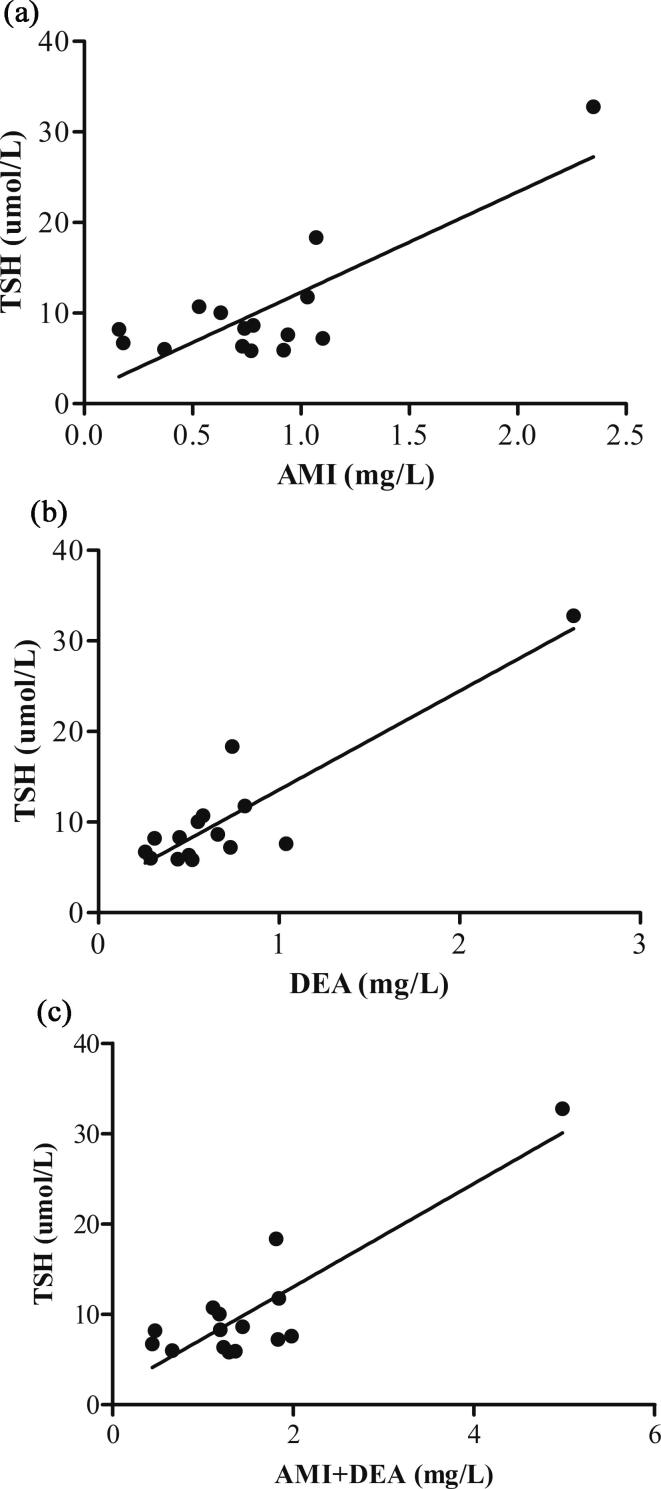
**a-c)** Correlation between elevated TSH (umol/L) and amiodarone (AMI), desethylamiodarone (DEA) and amiodarone + desethylamiodarone (AMI + DEA) serum concentrations. (a) Correlation between elevated TSH (umol/L) and AMI serum concentrations (mg/L) (number = 15; coefficient of correlation = 0.3536; p = 0.1961; y = 12.166x; R^2^ = 0.6606). (b) Correlation between elevated TSH (umol/L) and DEA serum concentrations (mg/L) (number = 15; coefficient of correlation = 0.6464; p = 0.0092; y = 13.244x; R^2^ = 0.7401). (c) Correlation between elevated TSH (umol/L) and AMI + DEA serum concentrations (mg/L) (number = 15; coefficient of correlation = 0.3536; p = 0.1961; y = 6,442x; R^2^ = 0,7467).

The adverse effects of amidarone therapy in the study group are discussed in Table 5. We have observed significant correlation between DEA serum concentration and elevation of TSH (p = 0.0092, coefficient of correlation = 0.6464). However, correlation was not observed in the event of AMI concentration (p = 0.1961, coefficient of correlation = 0.3536) and sum of AMI + DEA concentration (p = 0.1961, coefficient of correlation = 0.3536), see Fig. 2a-c.

**Table 5:** Adverse effects of amiodarone therapy during out-patient clinic visits.

**Adverse effects**			
**type of adverse effects**	**number of patients**	**AMI serum concentration (mg/L)/ *TSH (umol/L)***	**DEA serum concentration (mg/L)/ *TSH (umol/L)***
elevation of TSH (>5.6 umol/L)	14 (21%)	0.16/ *8.22*	0.31/ *8.22*
		0.18/ *6.72*	0.26/ *6.72*
		0.37/ *6.01*	0.29/ *6.01*
		0.53/ *10.73*	0.58/ *10.73*
		0.63/ *10.04*	0.55/ *10.04*
		0.73/ *6.35* (0.78/ *8.65*; 1.07/ *18.35*)	0.50/ *6.35* (0.66/ *8.65*; 0.74/ *18.35*)
		0.74/ *8.31*	0.45/ *8.31*
		0.77/ *5.84*	0.52/ *5.84*
		0.92/ *5.93*	0.44/ *5.93*
		0.94/ *7.61*	1.04/ *7.61*
		1.03/ *11.79*	0.73/ *7.23*
		1.10/ *7.23*	0.81/ *11.79*
		2.35/ *32.78*	2.63/ *32.78*
		not measured	not measured
elevation of liver enzymes	4 (6%)	0.65	0.80
		1.05	0.80
		1.14	0.97
		2.17	1.23
prolonged QTc interval on ECG (males > 450 ms females > 470 ms)	2 (3%)	1.0	0.43
		0.53	0.58
AV block	1 (1%)	1.0	0.63
sick sinus syndrome	1 (1%)	not measured	not measured
newly implanted dual chamber pacemaker	1 (1%)	not measured	not measured
color vision disorders	1 (1%)	0.54	0.54
dystrophia corneae verticillata	1 (1%)	not measured	not measured

## 5 Discussion

To our knowledge, this was the first study following serum concentration of AMI and DEA in patients after SA of AF. Moreover, our study presents a succes rate with systematic follow up according to a consensus statement on catheter and SA of AF from 2017 by the HRS/EHRA/ECAS/APHRS/SOLAECE (Calkins et al., 2017).

Our one-year succes rate is primarily based on 12-lead ECG and 24-hour Holter ECG monitoring. Although the general agreement is that early reccurence does not necessarily indicate long-term failure (Altman et al., 2014), prophylactic AMI treatment is recommended to reduce early arrhythmia reccurence after SA of AF. Its discontinuation by 3 months for patients in SR is warranted (Ad et al., 2016). However, routine TDM of AMI is usually not performed. We have noticed a high success rate in maintaining SR and thus confirmed the efficacy of AMI and DEA at the measured concentrations. In our study group, AMI helped to maintain SR in 83% of patients approximately 1 month after surgery and in 87% of patients for about 3 months after SA. This success rate was higher compared to the study from the same workplace but without therapy optimalization by TDM (71% and 80% of patients with SR, respectively) (Kolek and Brat, 2010). The majority of patients (91%) who didn't maintain SR had suffered from non-paroxysmal AF, which is in consistent with other studies in which non-paroxysmal AF is one of the predictors of late reccurrences (Kirchhof et al., 2016).

We have not demonstrated significant differences in serum concentration of AMI, DEA, sum of AMI + DEA concentration and DEA/AMI concentration ratio between patients with currently present SR and supraventricular tachyarrhythmia during the reference period except for the second outpatient visit, in which AMI, DEA and sum of AMI + DEA concentration were significantly higher in patients with supraventricular tachyarrhythmia. Simultaneously, the DEA/AMI concentration ratio was significantly lower in this patient subgroup. Although the dose of AMI was enhanced in some of the patients with persistent supraventricular tachyarrhythmia after the first outpatient visit and consequently serum concentrations of AMI and DEA were increased, these patients were still not able to convert to SR, suggesting that an increase of serum concentration above 1.0 mg/L couldńt lead to conversion to SR, as we expected. A lower DEA/AMI concentration ratio in these patients was probably caused by the failure to reach steady-state shortly after dose escalation. In that group with supraventricular tachyarrhythmia, 5 of 6 patients had preoperative non-paroxysmal AF which is a predictor of reduced SA efficacy. At the next outpatient visit (approximately 6 months after SA) 5 of the previous 6 patients converted to SR (4 of them after DC cardioversion), which may indicate that a longer time period is needed to left atrial structural reverse remodelling after SA of AF in these non-paroxysmal patients. Comparison with other studies is difficult because most of them dealt with serum concentration of AMI (and/or DEA) in observed patients who did not undergo a Maze procedure (or other type of ablation). Moreover, routine use of 24-hour Holter ECG monitoring after the ablation of AF in our study increases the percentage of patients with detected asymptomatic AF recurrences compared with performing only 12-lead ECG in some previous studies. Another question is, how exactly was drug assay applied in the previous studies, considering the fact that the lower limit of reliable quantitation set in the study by Rotmensch et al was 0.5 mg/L (Rotmensch et al., 1984). Nevertheless, similar results in a study by Saksena et at (Saksena et al., 1983) (refractory ventricular tachycardia), Greenberg et al Greenberg et al., 1987) (supraventricular and ventricular arrhythmia) and Haffajee et al (Haffajee et al., 1983) (refractory tachyarrhythmias) as well. On the other hand, serum concentration of AMI (and DEA) between 0.5 and 1.0 mg/L were measured in a majority of our patients with SR. Connoly et al (Connolly et al., 1988) observed that 67% of patients responded at an AMI concentration below 1.0 mg/L as well. Whereas previous studies analyzed serum concentration only retrospectively without any systematic attempt to optimize AMI therapy, Connoly et al determined the lowest concentration associated with an antiarrhythmic effect systematically by repeat heart rhythm monitoring (Connolly et al., 1988). Similarly, we tried to achieve the AMI concentration in which patients reached and maintained SR. Conversely, the effect of a higher serum concentration of AMI (>1.5 to 2.0 mg/L) was observed by Mostow et al in a group of patients with complex ventricular arrhythmias (Mostow et al., 1984). One of the reasons for this difference may be that ventricular arrhytmias may require higher serum concentrations of AMI compared with AF. Serum concentration of DEA seems to be more important than those of the parent compound for conversion of AF (Nielsen and Møller, 2000, Tieleman et al., 1997). At least 78% success was observed in our study group in patients with a DEA median serum concentration of 0.7 mg/L. One of the approaches for analysis of AMI and DEA serum concentration may be determining which patients are nonresponders to adequate serum concentrations. Therefore, if a patient does not respond to AMI therapy with a serum concentration of AMI (and DEA) in the range of 0.5–1.0 mg/L, it is unlikely that a therapeutic response will appear at a higher AMI dose (and higher serum concentrations). The recommended daily dose of AMI for maintaining SR according to the current recommendations is 100–200 mg once a day (after 10 g loading dose) (Gillis, 2016). The higher dosage used in our study aimed to at least achieve a lower limit of the recommended therapeutic range.

Adverse effects observed in our patients occurred at an AMI (and DEA) serum concentration in and below the lower limit of the recommended therapeutic range (0.5–2.5 mg/L) except for hypothyreosis. A substitution treatment of hypothyreosis had to be used in two patients. To our knowledge this was the first study in which correlation between DEA serum concentration levels and TSH was observed. The study by Yamato et al analyzed data sets from 330 patients and showed no significant relationship between the serum AMI and DEA concentrations and thyroid related hormone including TSH, fT4 and T3 (Yamato et al., 2017). Other authors had previously also reported the same finding (Lafuente-Lafuente et al., 2009). In terms of influence on thyroid function, only Anastasiou-Nana et al described a significant correlation between the AMI serum level and the rise of the rT3 serum level (Anastasiou-Nana et al., 1984). The effect of DEA was not examined in this study. The importance of DEA in this adverse reaction was also confirmed by Bogazzi et al who demonstrated that DEA, but not AMI, exerted a direct, although weak, effect on genes that are regulated by a thyroid hormone (Bogazzi et al., 2001). Similar to our study, thyroid dysfunction was observed at any AMI concentration in the study by Stäubli et al (Stäubli et al., 1983).

Prolonging of the QTc interval (in males > 450 ms, in females > 470 ms, Bazett's formula) was the reason for AMI dose reduction or treatment discontinuation in two patients. We have not observed any life-threatening adverse effects. Long-term tolerance of a higher dosage was also demonstrated by Kaski et al (Kaski et al., 1981) where patients who did not have recurrences had an average maintenance dose of 713 mg/day.

## 6 Limitations

Our current study has several limitations. It is a single-centre, prospective, observational study that analyzes long-term outcomes in patients with atrial fibrillation who have undergone SA of AF, and does not compare them with patients treated conservatively. It uses a small number of patients without universal continuous cardiac monitoring which could have led to overestimation of freedom from AF. On the other hand, incomplete follow-up could lead to underestimation of freedom from AF. A follow up of one year is a relatively short period but according to HRS/EHRA/ACAS consensus statements, all ablation trials should report efficacy at 12 months after the final ablation precedure. A potential source of errors may have been created when patients were discharged from the rigorously controlled clinical environment of the hospital to outpatient care. Athough patients were instructed not to take their morning medications on the study day, blood collection after administration cannot be excluded.

## 7 Conclusion

The main goal of the study was to try to determine the optimal therapeutic range for serum concentration of AMI and the pharmacologically effective metabolite DEA in the specific group of patients after SA of AF. Because of considerable interindividual variability of AMI (and DEA) serum concentration, analysis of these concentrations alone cannot replace assessment of the clinical response for treatment of individual patients. As with other antiarrhythmic agents, there appear to be patients whose arrhythmias do not respond to AMI regardless of the reached serum concentrations of the drug or metabolite. On the other hand, establishment of individual AMI (and DEA) concentrations at which optimal therapeutic response is achieved seems to be advantage. On this account, therapeutic monitoring of AMI and DEA should be helpful in personalised pharmacotherapy in patients after SA of AF.

## CRediT authorship contribution statement

**Hrudikova Erika:** Methodology, Data curation, Investigation, Writing - original draft. **Grundmann Milan:** Methodology, Writing - review & editing. **Kolek Martin:** Methodology, Data curation, Investigation, Writing - review & editing. **Urinovska Romana:** Investigation. **Kacirova Ivana:** Supervision, Methodology, Writing - review & editing.

## Declaration of Competing Interest

The authors declare that they have no known competing financial interests or personal relationships that could have appeared to influence the work reported in this paper.
